# A rare missense p.C125Y mutation in the *TNFRSF1A* gene identified in a Chinese family with tumor necrosis factor receptor-associated periodic fever syndrome

**DOI:** 10.3389/fgene.2024.1413641

**Published:** 2024-06-24

**Authors:** Mengqing Qian, Jingyu Zhou, Jing Wu, Haocheng Zhang, Shenglei Yu, Haoxin Xu, Yixuan Yang, Feiran Zhou, Qingluan Yang, Lingyun Shao, Wenhong Zhang, Ning Jiang, Qiaoling Ruan

**Affiliations:** ^1^ Shanghai Key Laboratory of Infectious Diseases and Biosafety Emergency Response, Department of Infectious Diseases, National Medical Center for Infectious Diseases, Huashan Hospital, Shanghai Medical College, Fudan University, Shanghai, China; ^2^ Shanghai Sci-Tech Inno Center for Infection and Immunity, Shanghai, China; ^3^ Department of Biostatistics and Computational Biology, State Key Laboratory of Genetic Engineering (SKLG), School of Life Sciences, Fudan University, Shanghai, China

**Keywords:** autoinflammatory disease, primary immunodeficiency disease, fever, tumor necrosis factor receptor-associated periodic syndrome, TNFRSF1A summary

## Abstract

**Background:**

Tumor necrosis factor receptor-associated periodic syndrome (TRAPS) is a rare autosomal dominant disorder with a low incidence in Asia. The most frequent clinical manifestations include fever, rash, myalgia, joint pain and abdominal pain. Misdiagnosis rates are high because of the clinical and genetic variability of the disease. The pathogenesis of TRAPS is complex and yet to be fully defined. Early genetic diagnosis is the key to precise treatment.

**Methods:**

In this study, a Chinese family with suspected TRAPS were analyzed by genome-wide SNP genotyping, linkage analysis and targeted sequencing for identification of mutations in causative genes. To study the pathogenicity of the identified gene mutation, we performed a conservation analysis of the mutation site and protein structure analysis. Flow cytometry was used to detect TNFRSF1A shedding and quantitative real-time PCR were used to assess the activation of unfolded protein response (UPR) in the mutation carriers and healthy individuals.

**Results:**

A typical TRAPS family history, with a pattern of autosomal dominant inheritance, led to the identification of a rare mutation in the *TNFRSF1A* gene (c.G374A [p.Cys125Tyr]) with unknown significance. The patient responded well to corticosteroids, and long-term therapy with colchicine effectively reduced the inflammatory attacks. No amyloid complications occurred during the 6-year follow-up. *In silico* protein analysis showed that the mutation site is highly conversed and the mutation prevents the formation of intrachain disulfide bonds in the protein. Despite a normal shedding of the TNFRSF1A protein from stimulated monocytes in the TRAPS patients with p.C125Y mutation, the expression of *CHOP* and the splicing of *XBP1* was significantly higher than healthy controls, suggesting the presence of an activation UPR.

**Conclusion:**

This is the first report of a Chinese family with the rare p.C125Y mutation in *TNFRSF1A*. The p.C125Y mutation does not result in aberrant receptor shedding, but instead is associated with an activated UPR in these TRAPS patients, which may provide new insights into the pathogenesis of this rare mutation in TRAPS.

## Introduction

Tumor necrosis factor receptor-associated periodic syndrome (TRAPS, MIM: 142680) is an autosomal dominant autoinflammatory disease caused by mutations in *TNFRSF1A*, which is located on chromosome 12p13 (McDermott et al., 1999). This gene encodes the tumor necrosis factor receptor (TNFRSF1A), which is widely expressed in a number of hematopoietic and non-hematopoietic cells (Idriss and Naismith, 2000). TNFRSF1A consists of an N-terminal signal peptide, an extracellular domain, transmembrane domain and C-terminal cytoplasmic domain (Cudrici et al., 2020).

The first case of TRAPS was documented in Ireland in 1982. In general, European populations have a higher incidence of the disease, when compared to Asian populations (Stojanov and McDermott, 2005). To date, 183 *TNFRSF1A* mutations have been reported in the Infevers database (http://fmf.igh.cnrs.fr/ISSAID/infevers/), with 44 missense mutations identified as pathogenic and 62 variations considered to be potentially pathogenic. Mutations in *TNFRSF1A* cause protein misfolding and retention in the endoplasmic reticulum (ER), thereby activating the unfolded protein response (UPR) (Lobito et al., 2006). The UPR is triggered by three ER transmembrane sensor proteins, PRKR-like endoplasmic reticulum kinase (PERK), activating transcription factor 6 (ATF6), and inositol-requiring protein 1α, which represent three different arms, and then change the state of the ER to adapt the stress such as accumulation of misfolded proteins (Chen et al., 2023). In addition to this hypothesis of TRAPS, increased reactive oxygen species production, impaired autophagy and increased production of pro-inflammatory factors are also implicated in the pathogenesis of the disease (Lobito et al., 2006; Kimberley et al., 2007; Simon et al., 2010; Bulua et al., 2011; Bachetti et al., 2013; Jarosz-Griffiths et al., 2019).

TRAPS is characterized by periodic fever, accompanied by a variety of non-specific symptoms (Romano et al., 2022). Renal amyloidosis is the most serious long-term complication but is rare in pediatric patients (Hodgson et al., 2014; Cody et al., 2021). Physical stress or emotion, mild infections, fatigue, hormonal changes, trauma and vaccinations can trigger the symptoms but the exact cause remains unknown (Li et al., 2023). Laboratory features that are typical of TRAPS, during febrile episodes, include elevated acute phase reactants, leukocytosis and thrombocytosis (Magnotti et al., 2013). A variety of treatment options are available, which not only reduce the acute symptoms but also prevent the chronic inflammation that leads to amyloidosis.

The disease typically manifests in early childhood, with a median age of symptom onset reported to be 4.3 years (Lachmann et al., 2014). However significant variation in the age of onset is observed in patients with TRAPS, with reports of disease onset or diagnosis in adulthood (Nakamura et al., 2009; Cantarini et al., 2014; Lopalco et al., 2015; Hernández-Rodríguez et al., 2016; Youngstein et al., 2018). There are limited reports of TRAPS in Chinese populations (Stojanov et al., 2004; Zhao et al., 2020; Li et al., 2023), with only nine adult cases documented in China and a median age of onset of 3 years (range: 0.5–38.5). In this study, we reported a Chinese patient with a family history of recurrent fever and abdominal pain, and identified a rare mutation in *TNFRSF1A*. To our knowledge, this is the first report of this mutation in the Chinese population.

## Methods

### Editorial policies and ethical considerations

This study was approved by the Ethical Review Committee of Huashan Hospital, Fudan University. All enrolled patients and non-affected family members who agreed to participate in this study provided written informed consent.

### Genome-wide single nucleotide polymorphism (SNP) genotyping and linkage analysis

**FIGURE 1:**
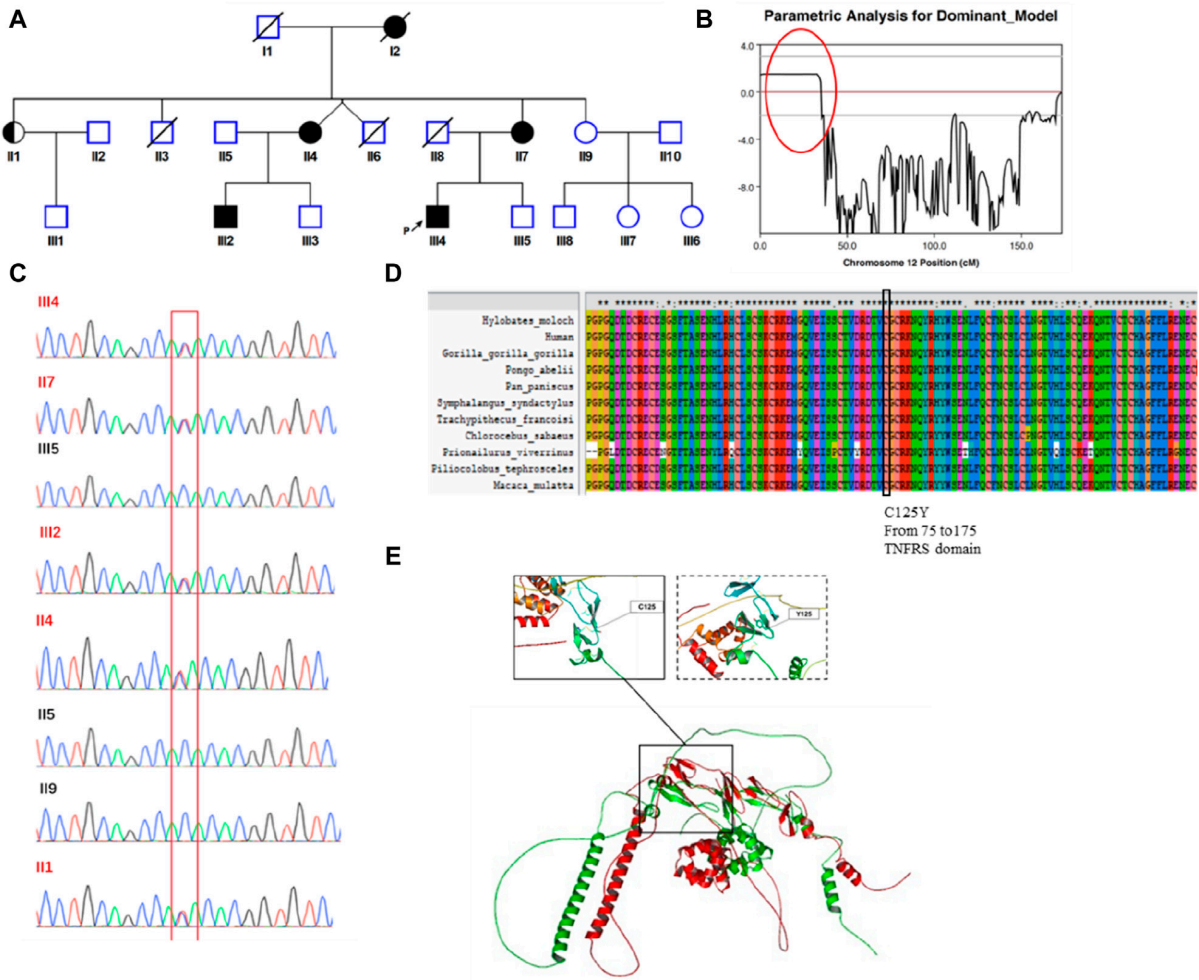
Identification of a germline missense mutation in *TNFRSF1A*. **(A)** Family Pedigree of the patient. The proband is indicated by a filled square with a black arrow. **(B)** Chromosome linkage analysis. A strongly linked segment was found on chromosome 12, marked with a red circle. **(C)** Sanger sequencing. The mutated *TNFRSF1A* gene (c.374G>A [p.Cys125Tyr]) was verified by Sanger sequencing in eight family members. **(D)** Conservation analysis. This mutation site was highly conserved across species. **(E)** Protein structure simulation. Structural alignment between the wild type protein structure and mutant protein. The left-hand box shows the normal structure surrounding the site of the wild type amino acid, with disulfide bonds present. The structure of the mutant protein is shown in the right-hand box, with no disulfide bonds.

Peripheral blood samples were collected from eight family members, denoted as II1, II2, II4, II5, II9, III2, III4 and III5 (Figure 1A), for genetic analysis. Genomic DNA was extracted using QIAGEN DNeasy Blood and Tissue kits (QIAGEN, Germany). Genome-wide genotyping was conducted using the Illumina GSA SNP chip, which includes 887,270 SNPs. The SNPs with “no-call”, allele frequency of <5% or those that did not match the parental genotype were excluded from the analysis. Subsequently, genome-wide SNP genotyping profiles were used for linkage analysis (five patients and three unaffected family members were analyzed). A total of 5,266 high-quality SNPs (one SNP per 0.5 cM) were included in the linkage analysis. Multipoint linkage analysis was conducted using MERLIN 1.1.2 software. Markers with the logarithm of odds score >1.5 were identified. Haplotypes were analyzed and visualized using the MERLIN 1.1.2 and HaploPainter software programs, respectively.

### Targeted panel sequencing and genotyping

We applied next-generation sequencing using a targeted panel of 535 genes to screen for primary immunodeficiency genes within this family. The biotinylated oligonucleotide probes specific to the exome regions of these 535 genes were designed by Roche^®^ NimbleDesign and manufactured by SeqCap^®^ EZ Prime Choice Probes. To minimize cost, two patients (III2 and III9) and one unaffected family member (III5) were selected for targeted panel sequencing using the Illumina Hiseq X-ten platform. The raw data (at least 1 Gb per sample/average 250 × mapping depth) were generated with a 150 bp paired-end read length. The adapter sequences and low-quality reads were filtered using trim-galore software. FastQC (http://www.bioinformatics.babraham.ac.uk/projects/fastqc/) software was used to evaluate the quality of the sequencing data. The remaining high-quality, clean reads were aligned to the human reference genome (hg19 version) using the Bowtie2 algorithm. The alignment results were sorted and marked PCR-duplicates using Picard. Only uniquely mapped reads were used for genotyping analysis. The SNPs and short insertions and deletions were identified using the GATK software. The ANNOVAR program was used for annotating mutations at both the gene and region levels. Sanger sequencing was conducted to confirm the variant of the gene using the following primers: *TNFRSF1A*-F: 5′- GGG​AAG​GAA​AGG​AAG​TGC​CA-3′, *TNFRSF1A*-R: 5′-AAT​GCC​GAA​AGG​GTG​AGT​GT-3’

### *In silico* protein analysis

To assess the level of conservation of the TNFRSF1A protein, the amino acid sequences of TNFRSF1A from humans and multiple other species were obtained from the NCBI protein database and analyzed using ClustalX v2 software (University College Dublin, Ireland). To assess the impact of the *TNFRSF1A* mutation on protein structure, we utilized the online AlphaFold2 website (https://colab.research.google.com/github/sokrypton/ColabFold/blob/main/AlphaFold2.ipynb) to predict the structures of both the wild-type and mutant TNFRSF1A proteins. The resulting protein structures were visualized by the Pymol software (The PyMOL Molecular Graphics System, Version 1.8.4.0.), using default parameters.

### Flow cytometry analysis of TNFRSF1A

Cryopreserved peripheral blood mononuclear cells (PBMCs) from the patient and his family members were thawed and allowed to rest in RPMI 1640, supplemented with 10% fetal bovine serum, for 6 h. The PBMCs were stimulated with 20 ng/mL of phorbol 12-myristate 13-acetate (PMA; BioGems, U.S.A.) at 37°C. Cells were collected after a 10 or 60 min stimulation and washed with ice-cold PBS buffer. After incubation with Fc Receptor Blocker (Biolegend, U.S.A.) for 15 min at 4°C, the cells were stained with PE-conjugated anti-human TNFRSF1A (Clone: W15099A) and FITC-conjugated anti-human CD14 (Clone: M5E2) antibodies (Biolegend, U.S.A.) for 30 min at 4°C. Stained cells were analyzed using CytoFLEX (Beckman Coulter, U.S.A), and the data were analyzed using FlowJo v10 (BD, U.S.A).

### Quantitative PCR analysis of UPR

Total RNA was extracted from peripheral blood using TRIzol reagent (Invitrogen, U.S.A.). The cDNA was synthesized using PrimeScript RT Master Mix (Takara, Japan). Quantitative real-time PCR was performed using the TB Green Premix Ex Taq II kit (Takara). The relative expression of spliced *XBP1* (*sXBP1*, F 5′-CTG​AGT​CCG​CAG​CAG​GTG-3′, R 5′-AGT​TGT​CCA​GAA​TGC​CCA​ACA -3′), *CHOP* (F 5′- CAGAACCAGCAGAGG TCACA-3′, R 5′- AGC​TGT​GCC​ACT​TTC​CTT​TC-3′) and *BIP* (F 5′- CAT​CAC​GCC​GTC​CTA​TGT​CG-3′, R 5′-CGT​CAA​AGA​CCG​TGT​TCT​CG-3′) in the TRAPS patients was calculated using the ΔΔC_t_ method, with *GAPDH* (F 5′-CTG​GGC​TAC​ACT​GAG​CAC​C-3′, R 5′-AAG​TGG​TCG​TTG​AGG​GCA​ATG-3′) serving as an internal control and unrelated individuals with wild-type TNFRSF1A serving as the control group.

### Statistical analysis

Data were represented using mean and standard deviation and analyzed using unpaired *t*-test with the GraphPad Prism nine software (Dotmatics, USA). *p* < 0.05 was considered statistically significant.

## Results

### Clinical features of the patient

On the 15th of April, 2018, a 29-year-old Chinese patient presented with fever and abdominal pain. Three weeks earlier, he had traveled to XinJiang and developed a fever of 40°C, with thoracalgia and muscular soreness. Two weeks before admission, he had developed right lower quadrant abdominal pain. There was tenderness and rebound tenderness on physical examination. The patient had an elevated white blood cell level of 14.52 × 10^9^/L, with 81% neutrophils, an elevated C-reactive protein level of 128.5 mg/L and an elevated IL-6 level of 9.4 pg/mL An abdominal CT scan revealed thickening of the wall of the ascending colon, accompanied by surrounding infiltration changes. The patient did not respond to potent empirical antibiotic therapy. After admission, small bowel obstruction was suspected because of dilated small bowel loops, with multiple stepwise fluid levels, on abdominal Xray. Routine stool and occult blood tests, plus a T-SPOT.TB assay, gave negative results. Screening tests for hepatitis virus, HIV and syphilis were also negative. A colonoscopy failed to reveal organic lesions in the terminal ileum and entire colorectal mucosa. There was no evidence of infection or malignant disease. The patient’s body temperature returned to normal 1 month after onset of the fever and all inflammatory markers returned to normal.

After examining the patient’s medical history in great detail, we found that he had experienced fever and abdominal pain approximately every 6 months since junior high school. He was diagnosed with appendicitis in 2003, due to significant acute lower quadrant pain. Following the appendectomy, he complained of discomfort in his abdomen and had a fever that peaked at 39°C. However, postoperative pathology showed no obvious inflammation. His fever and all other symptoms subsided after receiving empirical antibiotics for 3 days. Over the next 15 years, he experienced regular attacks of recurrent fever and abdominal pain, lasting more than 10 days and returning periodically on a semi-annual basis. Corticoids and immunosuppressive agents were never employed in the treatment of the event but non-steroidal anti-inflammatory drugs and empirical antibiotics were usually administered.

According to the patient’s family history, his mother (II 7), grandmother (I2), an aunt (II 4) and one elder male cousin (III 2) also suffered from recurrent fever (temperatures over 38°C, lasting for more than 3 days) with arthralgia or abdominal discomfort (Figure 1A). The female patients did not undergo systemic examination due to relatively mild symptoms, with inflammation that subsided spontaneously. His cousin underwent an exploratory laparotomy due to unknown “fever with abdominal pain” but ultimately no abnormalities were found.

### Identification of the germline missense mutation in *TNFRS1A*

The patient complained of recurring fever and abdominal discomfort, with no evidence of infection or malignant disease. In addition, he had a family history that was suggestive of a hereditary condition with autosomal dominant inheritance. Using samples from eight family members, we performed a genome-wide linkage analysis and discovered a significantly linked region on chromosome 12 (Figure 1B). Subsequently, we performed targeted next-generation sequencing of 535 primary immunodeficiency causative genes. We discovered 15 genes with non-synonymous single nucleotide variations (*CD46, NBAS, TGFBRAP1, C2, FGFR1, NUP214, DNLZ, ADRB1, TNFRSF1A, POLE, CIITA, STAT5B, ACE, LIG1KIR3DL3* and *RTEL1*), two with stop-gain mutations (*ENTPD1* and *BTNL2*) and one with a splice site mutation (*TTN*). The allele frequencies of those variants are lower than 0.01 in the population databases. These databases consisted of the 1000 Genomes Project database, dbSNP, the Exome Aggregation Consortium and the Genome Aggregation Database. On chromosome 12, there were only two genes: *POLE* and *TNFRSF1A*. Based on the patient’s clinical manifestations, we excluded the *POLE* mutation from being the suspected cause, and located the causative mutation on the *TNFRSF1A* gene (c.374G>A [p.Cys125Tyr]). Family members II1, II4, II7 and III2 also carried the mutation. Sanger sequencing verified the presence of the mutation (Figure 1C). This mutation was predicted to be pathogenic by multiple *in silico* pathogenicity prediction tools, such as CADD (score = 32), REVEL (0.992, pathogenic), SIFT (0, deleterious), PolyPhen-2 (1.0, probably damaging) and MutationTaster (0.88, disease causing). An analysis of interspecies conservation showed that there is a high degree of conservation of the amino acid at this site in the protein (Figure 1D). We then implemented PyMol for protein structure analysis and simulated the altered protein’s molecular structure. We discovered that the original location aids in the formation of disulfide bonds in the protein and the mutation may result in the absence of this process. When we analyzed the structure of the protein, we found that mutations can result in structural anomalies (Figure 1E). Most missense variants affected cysteine residues that are involved in disulfide bond formation (Aksentijevich et al., 2001; Rebelo et al., 2006). Lineage analysis revealed that changes in cysteine residues, which interrupt normal disulfide bond formation, result in more severe clinical phenotypes (Akagi et al., 2022). Notably, all carriers experienced recurring fever, except for individual II1, who remained asymptomatic. With an 80% penetrance rate in this family, it was hypothesized that the p.C125Y mutation exhibits incomplete penetrance and variations exist in the manifestation and severity of the clinical symptoms in p.C125Y mutation carriers.

### Normal shedding of TNFRSF1A from stimulated monocytes in patients with p.C125Y *TNFRSF1A* mutation

**FIGURE 2:**
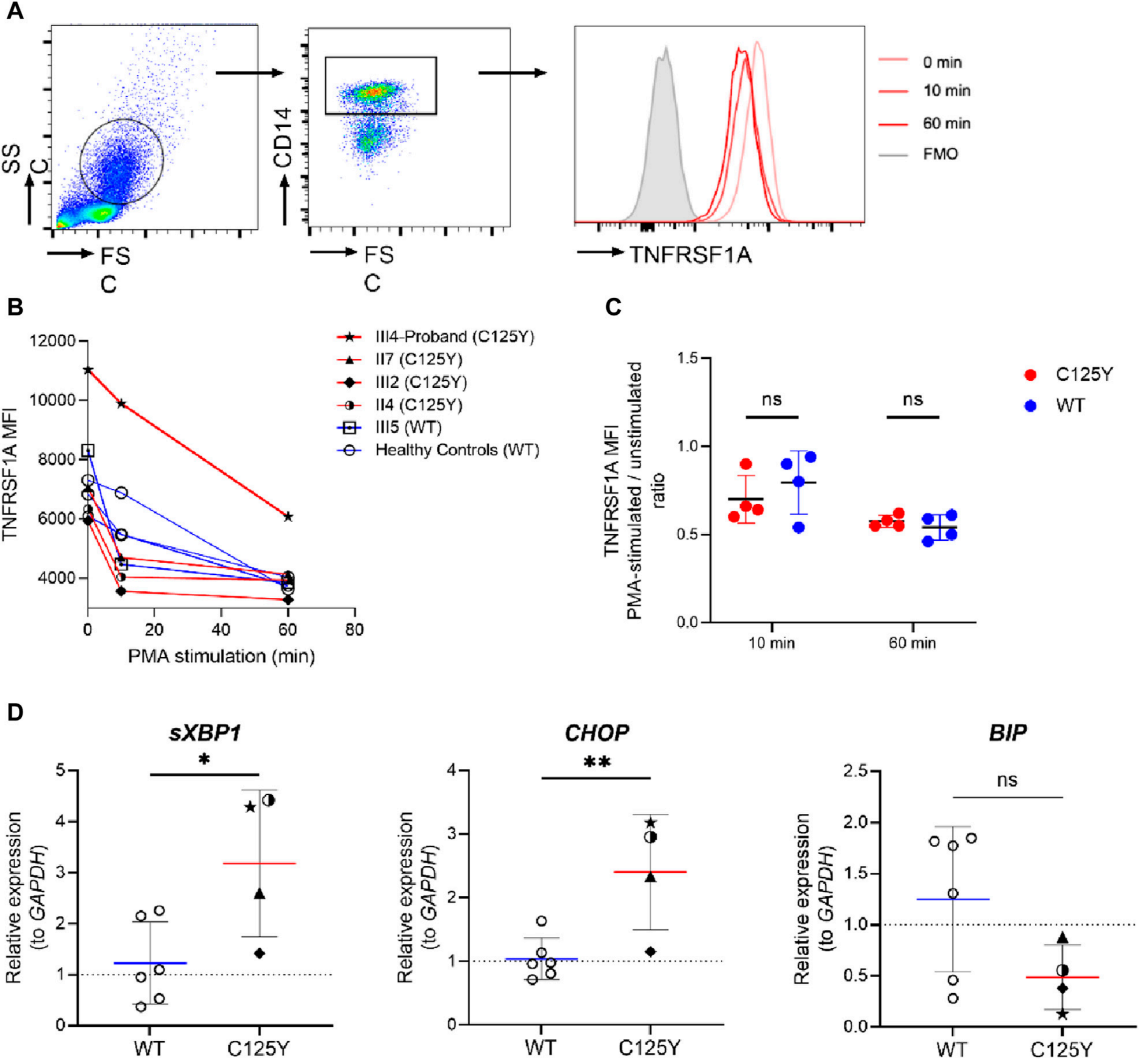
TNFRSF1A shedding and the activation of unfolded protein response (UPR) in patients with tumor necrosis factor receptor-associated periodic syndrome (TRAPS). **(A)** Gating strategy and representative histograms of TNFRSF1A level in a patient with the mutation. **(B)** Change in TNFRSF1A median fluorescence intensity (MFI) in peripheral CD14^+^ monocytes during 20 ng/mL phorbol 12-myristate 13-acetate (PMA) stimulation in patients with the mutation (red lines, n = 4), a family member with wild type (WT) *TNFRSF1A* (square icon, blue line, n = 1) and healthy controls (circle icon, blue line, n = 3). **(C)** Change in TNFRSF1A level in peripheral CD14^+^ monocytes after 10-min and 60-min PMA stimulation (MFI of PMA-stimulated cells/unstimulated cells ratio), in patients with the mutation (n = 4) and individuals with WT *TNFRSF1A* (n = 4). **(D)** The relative mRNA expression of spliced *XBP1* (*sXBP1*, left), *CHOP* (middle) and *BIP* (right) in TRAPS patients with the p.C125Y mutation (n = 4) and individuals with WT *TNFRSF1A* (n = 6) were assessed by quantitative real-time PCR. Results were analyzed using the ΔΔC_t_ method, with *GAPDH* serving as an internal control and unrelated individuals with WT *TNFRSF1A* serving as the control group. Data are shown as mean ± SD and compared using unpaired *t*-test. *, *p* < 0.05. **, *p <* 0.01.*ns*, not significant.

Proteolytic cleavage of TNFRSF1A is mediated in part by metalloproteases (Silke and Brink, 2010). Impaired surface TNFRSF1A shedding has been observed and is associated with dysregulated inflammation in TRAPS patients (McDermott et al., 1999). To assess whether the p.C125Y mutation in TNFRSF1A is associated with impaired shedding, PBMCs from the patient, family members and healthy controls were stimulated with PMA, which is a potent inducer of the metalloproteases that mediate receptor shedding (McDermott et al., 1999). The levels of TNFRSF1A in CD14^+^ monocytes were then determined (Figure 2A). In TRAPS patients with the p.C125Y mutation, the level of TNFRSF1A decreased in CD14^+^ monocytes during PMA stimulation. However, the decrease was comparable with healthy controls and the family member (III5) without the mutation (Figures 2B, C). This result indicated that normal shedding of TNFRSF1A is observed in TRAPS patients with the p.C125Y mutation.

### Elevated activation of UPR in patients with p.C125Y *TNFRSF1A* mutation

Another highly plausible pathogenesis of TRAPS involves an activated UPR triggered by accumulation of TNFRSF1A mutant in the ER (Lobito et al., 2006). Cells subsequently respond to this stress by inducing gene expression to restore proper ER function (Pahl and Baeuerle, 1997). To investigate the presence of an activated UPR, we therefore assessed the transcription of UPR-associated genes *CHOP* and *BIP*, and the splicing of *XBP1* (Sicari et al., 2020; Sen Santara et al., 2023). As shown in Figure 2D, there was no significant difference in the transcript level of BIP in these TRAPS patients with the p.C125Y mutation and healthy controls. However, a significantly elevated expression of *sXBP1* and *CHOP* was observed in TRAPS patients with the p.C125Y mutation. These results indicated a strong correlation between the p.C125Y mutation and the activation of UPR, which could subsequently induce cascades of inflammatory reactions, and therefore provided a possible explanation for the underlying mechanism of this p.C125Y mutation in TRAPS.

### Follow-up of the patients

After the patient received a definitive diagnosis, methylprednisolone, at a dosage of 24 mg per day, was prescribed to alleviate the acute symptoms. In addition, the patient was prescribed colchicine to reduce the episode attacks. He took the medication intermittently for several years and he occasionally experienced an episode attack. Over a 6-year follow-up, there was no evidence of secondary renal amyloidosis. His mother, two aunts and cousin did not take colchicine because of the low frequency of their episode attacks. The disease tended to alleviate with age.

## Discussion

The prominent clinical features of our case were recurrent fevers, each lasting at least 7 days, accompanied by obvious abdominal pain. These symptoms occurred once every 6 months. Inflammatory indicators increased significantly during the attacks and treatment with methylprednisolone significantly controlled the symptoms in the acute phase. Genetic testing revealed that the patient had a c.G374A (p.C125Y) mutation in exon four of the *TNFRSF1A* gene. This mutation has been recorded in a large case cohort and in a sporadic case report, with no family history or functional analysis (Dodé et al., 2002; Lachmann et al., 2014). This study demonstrated the process of diagnosing TRAPS in a patient with a 15-year history of recurrent fever, and a preliminary functional analysis was performed. To our knowledge, this is the first Asian case of this mutation being detected in adulthood.

There were no obvious abnormalities in the shedding of cell surface receptors, caused by the mutation, after PMA stimulation. This was consistent with previous studies by Mary L, Huggis, who observed that shedding was normal in individuals with amino acid changes such as p.C33Y (Huggins et al., 2004). The original pathogenic mechanism was thought to involve mutations that result in a receptor shedding disorder, causing a persistent inflammatory phenotype. However, shedding deficiency disorders display significant inter-patient variability (Aganna et al., 2003; Churchman et al., 2008; Greco et al., 2015).

We found the increased mRNA expression of *sXBP1* and *CHOP* in patients. Unconventional splicing of *XBP1* results from phosphorylation of inositol-requiring protein α, which activates transcription factors to be expressed (Calfon et al., 2002). *CHOP* is one of the important target downstream genes of the PERK arm, reflecting the activation status of this pathway (Avivar-Valderas et al., 2011). Besides, ER chaperone BiP/GRP78 can bind ATF6 and dissociate in response to ER stress (Shen et al., 2002). Our findings strongly suggest that this mutation activates inositol-requiring protein α arm and PERK arm, and it may have nothing to do with the ATF6 pathway. UPR can activate the mitogen-activated protein kinase and mitochondrial reactive oxygen species, then induce the secretion of pro-inflammatory cytokines, such as IL-1 and IL-6, to cause constitutive inflammation (Bulua et al., 2011). Our patient had elevated IL-6 levels, consistent with this possible causative mechanism. Autophagy dysfunction (Yang et al., 2015), excessive activation of NF-κb transcription factors and apoptosis suppression are also thought to play a role in the pathophysiology (Churchman et al., 2008; Greco et al., 2015). Indeed, there is a chance that these anomalies are related to one another, posing obstacles to our understanding of the relationships between genotype and phenotype. More research is needed to fully understand the functional consequences of this mutation.

TRAPS patients exhibit an extensive spectrum of clinical symptoms at onset and the disease is unquestionably genetically heterogeneous (Gaggiano et al., 2020). There is limited evidence that age or genotype significantly impact the disease characteristics at presentation (Lachmann et al., 2014). The disease is characterized by periodic fever, usually lasting 10–14 days, with a variety of non-specific symptoms such as prolonged or recurrent abdominal pain (70%), arthralgia (69%), myalgia (69%), swimming erythematous rash (60%), eye inflammation (37%) and periorbital edema (28%) (Cudrici et al., 2020). Amyloidosis, recurrent pericarditis, pleurisy, peritonitis and testicular inflammation are also possible complications. Previously, it was considered that a rash was more common among Chinese patients, when compared to European and Japanese patients, whereas chest pain was relatively uncommon (Zhao et al., 2020). However, the patient in this study had a 15-year medical history but lacked rash, which may change the distribution of clinical symptoms in the Chinese population. Recurrent abdominal pain also constitutes a clinical indication of TRAPS that requires more attention. More than one patient has been through an appendectomy and laparotomy due to signs of acute abdominal pain (Chen et al., 2014). Early diagnosis can eliminate the need for invasive procedures. For Chinese individuals, the average diagnostic delay is approximately 16.5 years and there is only an 11.1% possibility of having a positive family history (Zhao et al., 2020). Our patient had a family history and the diagnosis was established 15 years after the disease first emerged. The family history ruled out the possibility of a somatic mutation.

This pedigree had incomplete penetrance, which is an intriguing finding. Specifically, one carrier of the variant did not show any clinical features of TRAPS. There have also been reports of partial penetration with other variants in the past (Aksentijevich et al., 2001; Hull et al., 2002). The guidelines distinguish TRAPS mutations as high-penetrance or low-penetrance variants that are either pathogenic or variants of uncertain significance (Shinar et al., 2012). Luca Cantarini et al. compared the clinical manifestations and treatment responses between high-penetrance and low-penetrance variants. They suggested that low penetrance may be related to the autoinflammatory phenotype (Cantarini et al., 2014). Therefore, the phenomenon of incomplete penetrance in autosomal dominant diseases is a noteworthy and important aspect to consider.

We controlled the patient’s paroxysm with glucocorticoids, with a satisfactory outcome. Colchicine was used to prevent attacks. Corticosteroids and nonsteroidal anti-inflammatory drugs have been widely used for the control of acute symptoms (Ter Haar and Frenkel, 2014). With the expansion of disease research, biological agents are also being used in the treatment of TRAPS. These include TNF blockers (Etanercept), IL-1β receptor antagonists (Anakinra, Canakinumab) and IL-6 blockers (Tocilizumab), which are thought to be advantageous for avoiding complications (Vaitla et al., 2011; Ter Haar et al., 2013; Gentileschi et al., 2017; Kuemmerle-Deschner et al., 2020; Gattorno et al., 2024). However, IL-1β blockers were not available in China at that time. In addition, colchicine monotherapy may be tested in patients with milder phenotypes and at a lower risk of developing reactive amyloidosis (Vitale et al., 2020). The monitoring procedure had an enormous preventative impact, as evidenced by a marked decrease in strikes and the absence of amyloidosis. However, complete remission may not be possible with a single biologic treatment, since different pathophysiology may be involved in specific variations. Therefore, personalized treatment is highly recommended.

## Conclusion

This is the first family with TRAPS to be identified with the p.C125Y *TNFRSF1A* mutation in China. The p.C125Y mutation does not result in aberrant receptor shedding, but instead is associated with an activated UPR in these TRAPS patients. This case enriches the spectrum of human gene mutations and deepens our understanding of monogenic genetic diseases. TRAPS is a rare disease and should be taken into consideration if patients demonstrate recurrent fever. Family history and genetic sequencing provide critical evidence for diagnosis. Early diagnosis, standardized treatment and effective symptom and complication management can lead to improved patient outcomes, such as an extended lifespan.

## Data Availability

The original contributions presented in the study are included in the article/Supplementary Material, further inquiries can be directed to the corresponding authors.
