# Supplementary material for: A rare missense p.C125Y mutation in the TNFRSF1A gene identified in a Chinese family with tumor necrosis factor receptor-associated periodic fever syndrome
Source: Front Genet. 2024 Jun 24;15:1413641. doi: 10.3389/fgene.2024.1413641 (PMC11228257; doi:10.3389/fgene.2024.1413641)
Supplement: Supplementary file 1 [file DataSheet1.docx]

**Table S1. Raw data of quantitative real-time PCR**

| **Group** | **Sample** | ***sXBP1***  **C_t_ value** | | ***CHOP***  **C_t_ value** | | ***BIP***  **C_t_ value** | | ***GAPDH***  **C_t_ value** | |
| --- | --- | --- | --- | --- | --- | --- | --- | --- | --- |
| TRAPS patients with the p.C125Y *TNFRSF1A* mutation | III4 | 25.72 | 25.80 | 22.21 | 22.06 | 23.65 | 23.63 | 18.15 | 18.12 |
|  | II7 | 29.50 | 29.63 | 25.66 | 25.67 | 23.89 | 23.95 | 21.22 | 21.22 |
|  | III2 | 29.24 | 29.34 | 25.82 | 25.81 | 25.10 | 25.05 | 21.73 | 21.69 |
|  | II4 | 32.58 | 31.84 | 28.30 | 28.61 | 26.76 | 27.06 | 22.98 | 23.00 |
| Individuals with wild-type *TNFRSF1A* | Control1 | 26.32 | 26.25 | 23.25 | 23.62 | 19.87 | 19.86 | 17.78 | 17.69 |
|  | Control2 | 27.58 | 27.49 | 23.94 | 23.81 | 20.58 | 20.56 | 18.88 | 18.95 |
|  | Control3 | 28.57 | 28.13 | 24.07 | 24.01 | 20.25 | 20.12 | 18.57 | 18.54 |
|  | Control4 | 28.71 | 28.59 | 23.89 | 23.59 | 19.70 | 19.71 | 17.97 | 18.06 |
|  | Control5 | 28.70 | 28.50 | 23.52 | 23.33 | 21.35 | 20.82 | 17.31 | 17.58 |
|  | Control6 | 28.89 | 29.9 | 25.45 | 26.47 | 23.85 | 24.45 | 19.68 | 19.93 |
